# Autoantibody Profiling in Plasma of Dengue Virus–Infected Individuals

**DOI:** 10.3390/pathogens9121060

**Published:** 2020-12-18

**Authors:** Hoa Thi My Vo, Veasna Duong, Sowath Ly, Quan-Zhen Li, Philippe Dussart, Tineke Cantaert

**Affiliations:** 1 Immunology Unit, Institut Pasteur du Cambodge, Institut Pasteur International Network, 5 Monivong Blvd., Phnom Penh 12201, Cambodia; tvo@pasteur-kh.org; 2 Virology Unit, Institut Pasteur du Cambodge, Institut Pasteur International Network, 5 Monivong Blvd., Phnom Penh 12201, Cambodia; dveasna@pasteur-kh.org (V.D.); pdussart@pasteur-kh.org (P.D.); 3 Epidemiology and Public Health Unit, Institut Pasteur du Cambodge, Institut Pasteur International Network, 5 Monivong Blvd., Phnom Penh 12201, Cambodia; lsowath@pasteur-kh.org; 4 Department of Immunology and Internal Medicine, University of Texas Southwestern Medical Center, 5323 Harry Hines Blvd., Dallas, TX 75390, USA; Quan.Li@UTSouthwestern.edu

**Keywords:** dengue, autoantibody, infectious disease

## Abstract

Dengue is an arboviral disease caused by dengue virus (DENV) with high prevalence in tropical and sub-tropical regions. Autoimmune syndromes following dengue can be observed in long term follow up. Anti-DENV antibodies are cross-reactive with surface antigens on endothelial cells or platelets and could be involved in the pathogenesis of dengue. However, no studies have analyzed the autoantibody repertoire and its roles in dengue pathogenesis. Hence, we aimed to describe the autoantibody profile in dengue patients with different disease severities. We utilized a protein array with 128 putative autoantigens to screen for IgM and IgG reactivity in plasma obtained from healthy donors (*n* = 8), asymptomatic individuals infected with DENV (*n* = 11) and hospitalized dengue patients (*n* = 21). Even though the patient cohort is small, we show that 80 IgM and 6 IgG autoantibodies were elevated in DENV infected patients compared to age-matched healthy donors. Individuals undergoing a primary DENV infection showed higher amounts of IgG autoantibodies, not IgM autoantibodies, compared to individuals undergoing secondary infection. No differences were observed between asymptomatic and hospitalized dengue patients. Nineteen autoantibodies, which react against several coagulation and complement components, correlated with platelet counts in severe dengue patients. This current study provides a framework to explore a possible role of candidate autoantibodies in dengue immunopathogenesis.

## 1. Introduction

Dengue virus (DENV) is a member of the family Flaviviridae, and consists of four distinct DENV serotypes, DENV-1 to DENV-4 [1]. An estimated 390 million individuals are infected by DENV each year, of which ~25% are symptomatic [2]. Disease severity ranges from inapparent infection to classic dengue fever (DF) to dengue hemorrhagic fever/dengue shock syndrome (DHF/DSS). DHF/DSS occurs almost exclusively in patients re-infected with a different dengue serotype where hemorrhage, thrombocytopenia, vascular leakage and shock are the major clinical signs and possible cause of death in those patients [3,4]. Currently, no specific treatment is available for dengue.

Proposed mechanisms to explain immunopathogenesis include the development of a skewed T cell responses leading to cytokine storm and/or the existence of pre-existing, non-neutralizing antibodies causing antibody-depending enhancement [5]. During the acute phase of infection, a rapidly expanding plasmablast population has been observed, where plasmablast frequencies are increased compared to other infectious diseases or after influenza or yellow fever vaccination [6,7]. In addition, frequencies of circulating plasmablast are associated to more severe dengue disease [8]. Here, DENV is capable to activate B cells directly inducing polyclonal and highly cross-reactive antibody generation after both primary and secondary infection [9,10,11,12]. In steady state, polyreactive and autoreactive B cells are deleted from the mature naïve B cell repertoire at discrete checkpoints; however, these checkpoints could be defective in patients infected with DENV [13,14]. Indeed, a deregulated T cell compartment and increased circulating cytokine concentrations as observed during acute DENV infection could lead to the development of autoreactive antibodies [15,16]. This increased plasmablast population observed during DENV infection may contribute to the production of low affinity, cross-reactive and possible autoreactive antibodies.

Infection with Zika virus (ZIKV), a flavivirus closely related to DENV, has been reported to be associated with Guillain-Barre syndrome, an autoimmune disease caused by anti-ganglioside and anti-glycolipid antibodies [17,18,19,20,21]. Compared to healthy donors, patients infected with DENV had an overall higher risk to develop autoimmune diseases during long-term follow up such as multiple sclerosis, myasthenia gravis, autoimmune encephalomyelitis, systemic vasculitis, systemic lupus erythematosus, and others [22]. In contrast, a recent study in Taiwan observed a lower risk of primary Sjogren’s syndrome in previous patients infected with DENV [23]. Two years after infection, patients with long-term persistence of symptoms showed altered autoimmune markers compared to asymptomatic infected individuals [21]. Thus, accumulating evidence suggest that DENV infection may have long-term effects priming individuals to autoimmune diseases.

A variety of autoantibodies against host factors such as endothelial cells, platelets and components in coagulation pathways were observed in plasma of patients infected with DENV during the acute phase and up to 8 months after infection [24,25,26,27]. Plasma from patients infected with DENV could enhance activation of endothelial cells and platelets resulting in disturbing their physiological functions [28,29]. Indeed, increased anti-platelet autoantibody levels in patients infected with DENV were associated with acute secondary infection [30] and dengue severity [27]. Moreover, autoantibodies against viral non-structural protein 1 (NS1) are detected early after DENV infection and NS1-specific antibodies are cross-reactive with blood clotting proteins and endothelial cell antigens, and are hypothesized to contribute to manifestation in DHF/DSS patients [24,29,31,32]. Recently, it has been shown that both patients infected with DENV and DENV NS1-immunized rabbits produced antibodies against TACI, BCMA, and BAFFR, which are receptors expressed on B cells important for B cell maturation and survival. In a dengue mouse model, these antibodies suppressed DENV neutralizing antibody formation [33].

Taken together, it has been suggested that autoantibodies could be involved in the pathogenesis of dengue; however, a systematic screen of autoantibody reactivities during the acute phase of DENV infection has not been performed. Therefore, we utilized a proteomic microarray, which has the capacity to hold large number of self-antigens on a solid surface, to screen plasma from patients with different disease severity at early convalescence, 6–10 days after laboratory-confirmed DENV infection. Even though the patient cohort is small, we show that 80 IgM autoantibodies were elevated in patients infected with DENV compared to age-matched healthy donors, indicating general immune activation after viral infection. Presence of IgG autoantibodies against several components of the complement pathway such as Factor P and Complement C4, and coagulation pathways such as Prothrombin protein were correlated with platelet counts in DHF patients. These findings provide an exploratory analysis of autoantibody profiles in DENV infection and its association with disease severity.

## 2. Results

### 2.1. Characteristics of the Study Population

A total of 40 Cambodian children were included in the study. Of these, eight were healthy donors (HD) and 32 were individuals infected with DENV. DENV infection was confirmed by detection of viral RNA in the serum and measured as copies/mL (Table 1). Of the dengue patients, 21 patients were hospitalized and 11 individuals were undergoing asymptomatic infection (ASD). Here, asymptomatic individuals were identified via a household-based cluster investigation conducted around confirmed DENV-infected cases [34]. The demographic information and laboratory characteristics from the patients are summarized in Table 1. DENV serotypes were identified by serotype-specific reverse transcription-qPCR (RT-qPCR) and showed that DENV-1 was the most prevalent serotype. The patients, who were admitted at the hospital at 3 ± 2 days after the onset of symptoms, were classified as dengue fever (DF; *n* = 13) and dengue hemorrhagic fever (DHF; *n* = 8) according to the WHO 1997 guidelines [35]. Six individuals were undergoing primary infection and twenty-six individuals were undergoing secondary DENV infection. As expected, platelet counts were lower in patients with DHF compared to patients with DF (Table 1).

### 2.2. Autoantibody Profiles in DENV-Infected Patients

Antibodies against self-antigens such as platelets, endothelial cells and molecules from the coagulation pathway have been detected in patients infected with DENV [23,25,26]. In current study, an antigen array was used for high-throughput autoantibody profiling in patient plasma. The array consists of 123 putative autoantigens, containing a variety of proteins related to nuclear proteins, basement membrane proteins, cell stress-related proteins and others [36]. In the microarray, the binding of autoantibodies to each antigen spotted on nitrocellulose coated slide resulted in a fluorescence signal. The normalized fluorescence intensity (NFI) of each antigen was quantified and shown (Supplementary Figure S1). We analyzed plasma at 6–10 days after PCR-confirmed DENV infection, as we reasoned that at this time point sufficient amounts of potential autoantibodies would be present in circulation for detection.

**Figure 1:**
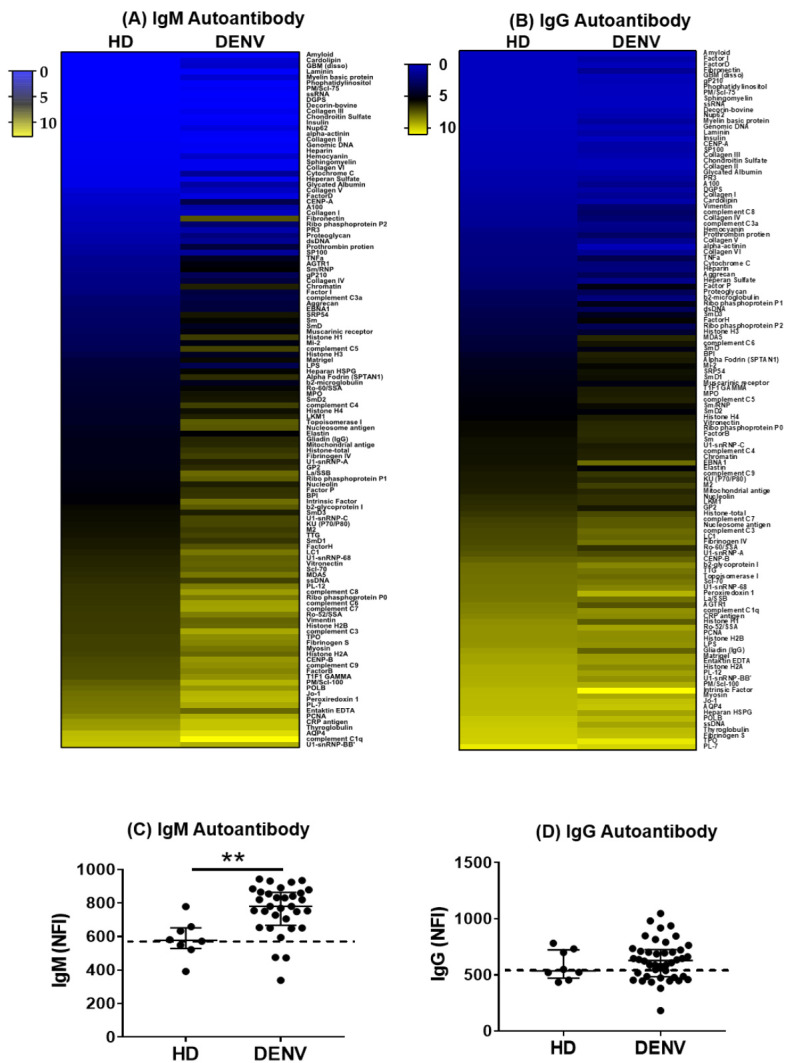
Distinct autoantibody profiles between healthy donors (HD) and DENV-infected individuals. (**A**,**B**) In the heatmaps, each row shows the average NFI of IgM or IgG autoantibodies of each group. The colors represent values of NFI of autoantibodies. (**C**,**D**) Each dot represents the sum of NFI signals of IgM autoantibodies or IgG autoantibodies in each individual. The dashed lines indicate the baseline of the median NFI in HD group. Statistical analysis was done using two-tailed Mann–Whitney tests to compare the two groups. Median and interquartile ranges are shown. ** *p* < 0.01. HD = 8, DENV = 32.

Viral infections are known to induce polyclonal B cell stimulation and bystander activation [10,37,38,39]. Therefore, we first investigated the presence of autoantibodies in patients infected with DENV compared to HD. Figure 1A,B displays the heatmaps where autoantibody expression is ordered from lowest to highest (blue: low expression, yellow: high expression). Significantly, higher NFI of IgM autoantibodies can be observed in patients infected with DENV compared to HD, whereas the NFI of IgG autoantibodies was comparable between both groups (Figure 1C,D). Furthermore, to distinguish the autoantibody profiles elicited in patients infected with DENV and HD, the NFI of each autoantibody was compared. Eighty IgM and six IgG autoantibodies were identified that had increased NFI in DENV-infected patient sera compared to HD (Supplementary Figure S2A,B). Among these eighty IgM autoantibodies, patients infected with DENV exhibited increased NFI against β2-glycoprotein-I (β2GPI) and protein components in the complement pathway such as C5, C8, C9 and factor B compared to HD. Interestingly, both IgM and IgG autoantibodies against factor H and factor P were present in patients infected with DENV compared to heathy controls. Taken together, our findings suggest that individuals produce a variety of autoantibodies, especially, IgM autoantibodies, shortly after DENV infection, which is probably due to bystander activation of circulating memory B cells [10,12,40,41].

### 2.3. Increased IgG Autoantibody NFI Are Associated with Primary DENV Infection

As immune status to DENV is an important determinant for the humoral response to DENV [7,41,42,43,44], we aimed to understand if a secondary infection would also lead to an altered production of autoantibodies. Therefore, we stratified our patient cohort in primary and secondary infected individuals, yielding only 6 individuals in the primary infected group. A limitation here is that subjects in the primary infected group were all male and infected by DENV-1, whereas in the secondary infected group only 42% were male and 73% were infected with DENV-1, which could bias the data.

**Figure 2:**
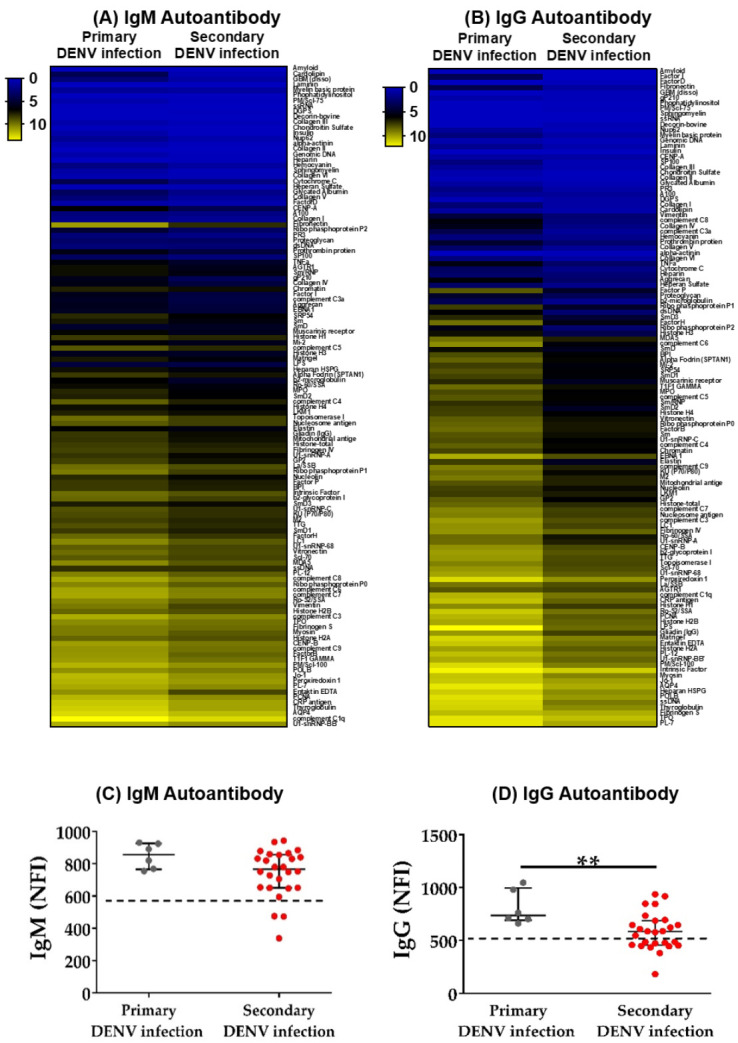
Comparison of expression of autoantibodies between primary and secondary DENV infection. (**A**,**B**) In the heatmaps, each row shows the average NFI of IgM or IgG autoantibodies in each group. The colors represent values of NFI of autoantibodies. (**C**,**D**) Each dot represents the sum of NFI signals of IgM autoantibodies or IgG autoantibodies in each individual. The dashed lines indicate the baseline of the median NFI in the HD group. Statistical analysis was done using two-tailed Mann–Whitney tests to compare two groups. Median and interquartile ranges are shown. ** *p* < 0.01. Primary DENV infection = 6, Secondary DENV infection = 26.

Here, the IgM and IgG autoantibody NFI was higher in primary infected patients compared to patients undergoing secondary infection (Figure 2A,B). When compared to the total NFI generated by each individual, the NFI of IgG antibodies in patients with primary infection was significantly higher than in patients with secondary infection (Figure 2C,D). Furthermore, to distinguish the autoantibody profiles, the NFI of each autoantibody was compared. Fifteen IgM and seventy IgG autoantibodies were identified that had increased NFI in primary DENV-infected patient sera compared to secondary infected sera (Supplementary Figure S3A,B).

### 2.4. Decreased Autoantibodies Correlate with Severity

**Figure 3:**
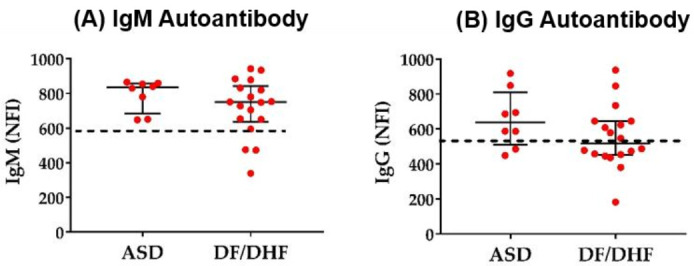
Autoantibody expression in secondary DENV infection. (**A**,**B**) Each dot represents the sum of NFI signals of IgM autoantibodies or IgG autoantibodies in each individual. The dashed lines indicate the baseline of the median NFI in HD group. Statistical analysis was done using two-tailed Mann–Whitney tests to compare the two groups. Median and interquartile ranges are shown. ASD = 8, DF/DHF = 18.

While primary DENV infections mostly lead to inapparent or mild disease symptoms, secondary infections can cause a wide range of disease symptoms from classical DF to DHF or DSS [45,46,47]. Hospitalized patients infected with DENV are characterized by higher serum anti-DENV IgM concentrations, activation of B cell response and increased plasma cell development compared to asymptomatic infected patients [34]. Hence, we aimed to investigate whether disease severity could be associated to the development of autoantibodies during infection. Therefore, IgG and IgM autoantibody profiles in patients with secondary DENV infection and with different disease severity classified according to WHO 1997 criteria were analyzed. No differences in the NFI of total autoantibodies were found between asymptomatic infected individuals and hospitalized patients (Figure 3A,B) or in DF patients compared to DHF patients (Supplementary Figure S5). However, analyzing the NFI of individual antibodies, we identified four IgM and fourteen IgG autoantibodies that showed decreased NFI in DF/DHF patients compared to asymptomatic infected individuals (Supplementary Figure S4A,B). Those antibodies bind to extracellular and intracellular matrix proteins such as heparan sulfate, proteoglycan and mitochondrial-associated proteins.

**Figure 4:**
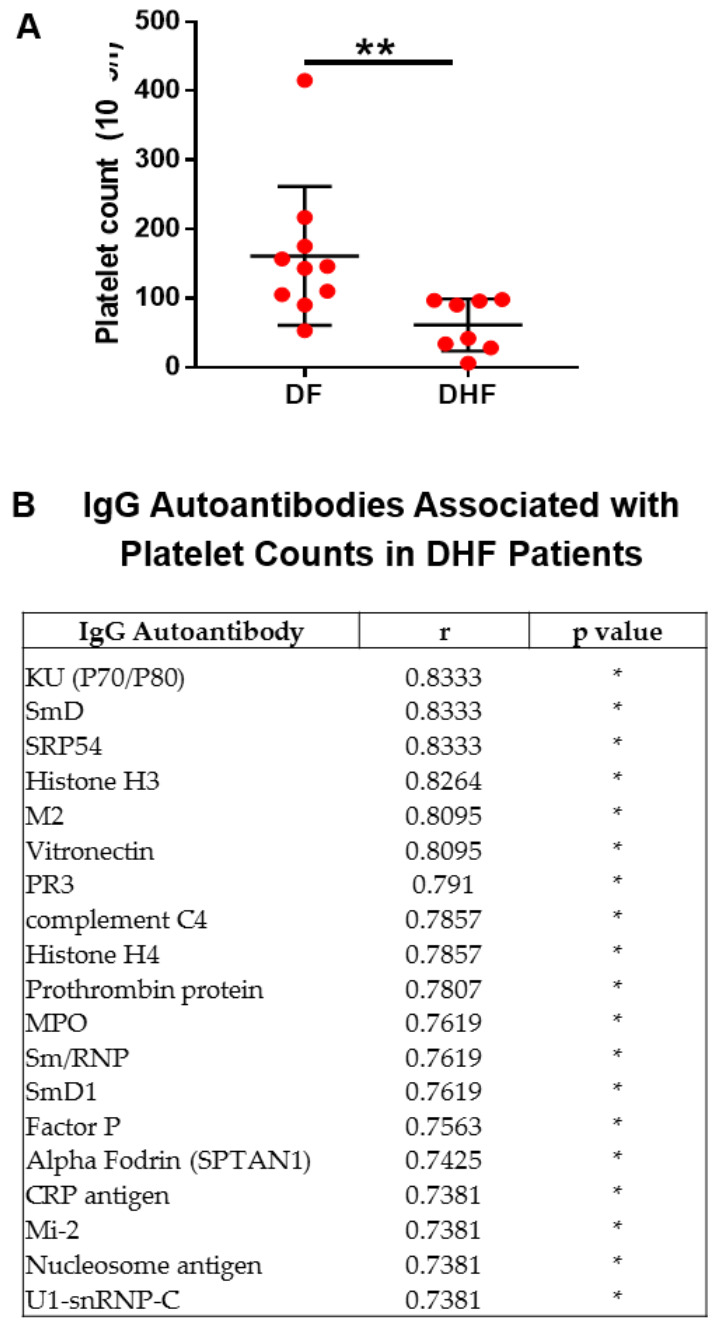
Autoantibody expression correlates with platelet counts. (**A**) The graph shows the platelet count in patients infected with DENV. Statistical analysis was done using two-tailed Mann–Whitney tests to compare the two groups. Median and interquartile range are shown. (**B**) The table presents the IgG autoantibodies in DHF patients that correlated with platelet count. Non-parametric Spearman test was used for correlation analyses. * *p* < 0.05, ** *p* < 0.01. DF = 10 and DHF = 8.

Thrombocytopenia is one of the key features of severe dengue disease. As expected, the platelet count in DHF patients was significantly lower compared to DF patients (Figure 4A). Among the 123 IgG autoantibodies, 19 of them positively correlated with platelet counts in DHF patients (Figure 4B). For example, it was observed that low amounts of anti-factor P IgG and anti-complement C4 were associated with low platelet counts in DHF patients. Other antibodies associated with platelet count are antibodies against nuclear proteins, such as KU (P70/P80) DNA-binding antigen, Smith antigen, histone antigen and nucleosome antigen. Taken together, these data suggest the presence of a subset of IgG autoantibodies in individuals infected with DENV that required hospitalization.

## 3. Discussion

In current study, we have screened 32 dengue-infected patients with variable disease severity for the development of autoantibodies in the early convalescent phase by protein array containing 128 autoantigens. Even though the patient cohort is small, we show that 80 IgM and 6 IgG autoantibodies were elevated in DENV infected patients compared to age-matched healthy donors. Individuals undergoing a primary DENV infection showed higher amounts of IgG autoantibodies, not IgM autoantibodies, compared to individuals undergoing secondary infection. No differences were observed between asymptomatic and hospitalized dengue patients. Nineteen autoantibodies, which react against several coagulation and complement components, correlated with platelet counts in severe dengue patients.

Various viral diseases induce transient autoantibodies during infection. The mechanism underlying the production of autoantibodies could be due to molecular mimicry, the presence of superantigens, epitope spreading and polyclonal activation due to the inflammatory milieu [48]. These antibodies could be directly involved in exacerbation or protection from severe disease or they could be mere a bystander effect of general immune activation and inflammation. They could have a role in possible clearance of virus and virus-infected cells, interfere with the binding of the virus to the host cell or could participate in the immune response through immune-complex formation and antigen clearance [48,49,50].

In patients infected with DENV, B cells show an activated phenotype leading to massive early plasmablast formation [8,11,51]. The current study demonstrates that eighty different IgM autoantibodies against complement components, coagulation factors and intracellular antigens were increased in patients infected with DENV compared to HD. These IgM autoantibodies may be generated from early-activated plasmablasts and could reflect bystander activation of the B cell compartment [10,37,39].

Serum-induced platelet lysis was observed in patients infected with DENV but not in patients infected with other members of the Flaviviridae family such as Japanese encephalitis virus and hepatitis C virus, or enterovirus A71 [27]. Here, we show the detection of IgM autoantibodies against platelets in patients infected with DENV. Interestingly, during DENV infection, IgM and IgG antibodies against NS1 protein can be detected which are cross-reactive with platelet antigens [32,52] and their presence correlate with disease severity [27,52].

We observed that patients infected with DENV exhibited higher levels of IgM autoantibody against β2GPI compared to HD, which is in parallel with findings in other infectious diseases such as in patients with HIV, syphilis, leprosy and hepatitis C [53] or in patients with autoimmune thrombotic disorder [54]. Anti- β2GPI IgG binds to cell surface molecules of endothelial cells, platelets and immune cells thereby resulting in cell activation [54]. Further studies are needed to clarify whether by targeting platelet cells and endothelial cells, β2GPI autoantibodies could contribute to dengue pathogenesis.

In the current study, a variety of autoantibodies targeting components of the complement pathway such as C5, C8, C9, factor B, factor H and factor P were detected in patients infected with DENV compared to HD. A robust activation of the complement pathways is suggested to contribute to damage of blood vessel cells and initiation of vascular leakage in DENV infection. While factor H inactivates the alternative C3 convertase (C3bBb) complex leading to inhibition of the alternative complement pathway, factor P has a role in stabilizing the complex, thereby enhancing activation of the alternative complement pathway [55]. It has been reported that compared to DF patients, DHF patients had a lower expression of factor H, following increased formation of C3bBb complex promoting the alternative complement cascade. IgG autoantibodies against factor H and factor P were present in patients infected with DENV compared to heathy controls. Furthermore, amounts of IgG autoantibodies against factor H and factor P were higher in individuals with primary infection than those in secondary infection. As expected, the platelet count was lower in DHF patients compared to DF patients. Interestingly, low amounts of anti-factor P IgG, anti-prothrombin IgG and anti-complement C4 IgG were associated with low platelet counts in DHF patients, whereas such association was not observed in DF patients. This might reflect an increased clearance of these autoantibodies through immune complex formation with C4 and factor P, contributing to the imbalance in the complement cascade, promoting DHF progression. In addition to complement and coagulation proteins, many autoantibodies targeting extracellular and intracellular matrix proteins such as heparan sulfate, proteoglycan and mitochondrial antigens were decreased in DF/DHF patients compared to individuals with ASD. Heparan sulfate moieties of proteoglycans are a putative receptor for DENV [56]. Therefore, anti-heparin sulfate antibodies might interfere with DENV attachment to cell membranes and thereby block subsequent infection.

Besides anti-factor P IgG and anti-complement C4, other IgG autoantibodies were found associated with platelet count in DHF patients. Those antibodies bind nuclear proteins, such as KU (P70/P80) DNA-binding antigen, Smith antigen, histone antigen and nucleosome antigen, which are prevalent in autoimmune disorders [57].

Secondary DENV infection induces a rapid increase in DENV-specific IgGs compared to primary infection [12,58]. Here, we detected higher amounts of IgG autoantibodies in individuals undergoing a primary DENV infection compared to individuals undergoing secondary infection. The presence of IgG autoantibodies reflects leakiness in tolerance mechanisms, allowing the maturation of autoantigen-binding B cells and their subsequent differentiation into antibody-secreting plasma cells [59].

The relationship between autoimmunity and infection has been debated for a long time. On one hand, the presence of autoimmunity can enhance infection. For example, patients with primary Sjogren’s syndrome showed 14% higher chance to experience hepatitis C infection than healthy controls; and those patients had a higher prevalence of hepatic manifestations [60]. On the other hand, many infection can cause the transient development of autoimmunity [48]. How long the detected autoantibodies last after the infection is unknown as we do not have follow-up samples of these individuals. Another caveat of our study is the limited sample size, therefore our study lacks power to detect small differences. In addition, due to the low amount of plasma available, we were unable to perform any cross-validation experiments. Therefore, further studies are still needed to address these remaining concerns and questions.

This exploratory study profiles developing autoantibodies in patients infected with DENV using autoantigen microarray. We observed that patients infected with DENV had higher levels of IgM autoantibodies than HD, indicating a general immune activation after viral infection. Higher amounts of IgG autoantibodies were detected in patients undergoing primary infection compared to secondary infection. Whereas no differences were observed between asymptomatic and hospitalized patients infected with DENV, reduced platelet count in severe patients infected with DENV was correlated to lower amounts of several autoantibodies. Current study provides preliminary data to further explore a possible role of candidate autoantibodies in dengue pathogenesis.

## 4. Materials and Methods

### 4.1. Ethics Statement

The ethical approval and recruitment of patients for this study has been described before [34,61]. Ethical approval for the study was obtained from the National Ethics Committee of Health Research of Cambodia. Written informed consent was obtained from all participants or the guardians of participants under 18 years of age before inclusion in the study.

### 4.2. Patient Samples

Hospitalized dengue cases were identified from patients presenting with acute dengue-like illness between June and October of 2012 and 2013 at Kampong Cham City Provincial Hospital and two district hospitals in Kampong Cham province. Plasma specimens were tested for DENV infection at the Institut Pasteur du Cambodge, the reference center for arboviral diseases in Cambodia. Patients were diagnosed as acute DENV-infected as following: a positive RT-qPCR [50] or NS1 positive by rapid test (SD Bioline Dengue Duo kits from Standard Diagnostics, Abbott, Chicago, IL, USA) at hospital admission, or seroconversion from DENV-IgM negative to IgM positive during the hospital stay (admittance and discharge sample). Platelet counts and hematocrit were determined by complete blood count at hospital admittance and patients were classified for severity according to WHO 1997 criteria upon discharge [35]. In total, 21 plasma samples from DENV-positive patients at hospital discharge were included in this study (Table 1). A cluster investigation was initiated, enrolling all family members in the household and people living within a 200-m radius of the home of the hospitalized dengue cases [61]. Here, individuals were diagnosed as acute DENV-infected by RT-qPCR at time of blood sampling. Individuals were questioned about history of symptoms 4 days before and were followed up until 10 days after sampling for the occurrence of symptoms (including but not limited to fever, rash, headache, retro-orbital pain). The blood samples from those individuals without any symptoms but positive for DENV detection were collected at day 0 (D0) as the day positive-DENV detection and day 7. In addition, age- and sex-matched negative DENV individuals (*n* = 8) from the cluster-based investigation were included as healthy control.

### 4.3. Protein Microarray

IgG and IgM autoantibodies against 123 antigens were measured using an autoantigen microarray platform developed by University of Texas Southwestern Medical Center (https://microarray.swmed.edu/products/category/protein-array/). Briefly, plasma samples were retreated with DNases-I to remove free DNA and then diluted 1:50 in PBST buffer for autoantibody profiling. The autoantigen array bearing 123 autoantigens and 4 control proteins were printed in duplicates onto Nitrocellulose film slides (Grace Bio-Labs). The diluted serum samples were incubated with the autoantigen arrays, and autoantibodies were measured with cy3-conjugated anti-human IgG (1:2000, Jackson ImmunoResearch Laboratories) and cy5-conjugated anti-human IgM (1:2000, Jackson ImmunoResearch Laboratories), using a Genepix 4200A scanner (Molecular Device) with laser wavelengths of 532 and 635 nm. The resulting images were analyzed using Genepix Pro 7.0 software (Molecular Devices). The median of the signal intensity for each spot was calculated and the background around the spot was subtracted, and data obtained from duplicate spots were averaged. The background subtracted signal intensity of each antigen was normalized to the average intensity of the human IgG or IgM, which were spotted on the array as internal controls. Finally, the normalized fluorescence intensity (NFI) for each antigen was calculated by subtracting a PBS control which was included for each experiment as negative control. Signal-to-noise ratio (SNR) was used as a quantitative measurement of the true signal above background noise. SNR values equal to or greater than three were considered significantly higher than background, and therefore true signals. The NFI of each autoantibody was used to generate heatmaps using Cluster and Treeview software (Version 3) (http://bonsai.hgc.jp/~mdehoon/software/cluster/software.htm). Each row in the heatmap represents an autoantibody and each column represents a sample. Yellow color represents the signal intensity higher than the mean value and blue color means signal intensity is lower than the mean value. Grey or black color indicates the signal is close or equal to the mean value of the raw.

### 4.4. Statistical Analysis

Data were analyzed and plotted using GraphPad Prism, version 7.0. The data were tested and did not pass the D’Agostino-Pearson normality test. Therefore, all group comparisons were performed with a non-parametric Mann–Whitney test or Chi2-test/Fisher’s exact test as indicated. Demographic and clinical continuous data were expressed as the mean ± SD while nominal data were presented as percentage (%) in the table. Median value and interquartile range of the data were shown in the scatter plots. Non-parametric Spearman’s method was used for correlation analyses. To compare the autoantibody patterns between the groups, the multiple t-test analysis was applied. Statistical significance was assumed at *p* < 0.05.

## Figures and Tables

**Table 1:** Demographic and clinical characteristics of the study population**.**

Characteristics	HD	ASD	DF	DHF	*p*-Value
	(*n* = 8)	(*n* = 11)	(*n* = 13)	(*n* = 8)	
Age (year)	8.9 ± 5.6	11.5 ± 3.1	9.9 ± 3.0	10.1 ± 2.9	0.4 ^a^
Sex (*n*, %)					0.3 ^b^
Female	4 (50%)	3 (30%)	7 (53.9%)	5 (62.5%)	
Day of Fever (day)	NA	NA	7.3 ± 0.8	7.1 ± 0.8	0.8 ^c^
Temp (°C)	NA	NA	37.6 ± 1	36.9 ± 0.4	0.1 ^c^
Pulse (bpm)	NA	NA	100 ± 15	96 ± 14	0.6 ^c^
Systolic Blood Pressure (mmHg)	NA	NA	100 ± 10	98 ± 10	0.8 ^c^
Diastolic Blood Pressure (mmHg)	NA	NA	63 ± 11	63 ± 9	0.8 ^c^
Hematocrit (%)	NA	NA	38.5 ± 3.9	43.1 ± 4.8	0.05 ^c^
Platelets (10^9/l)	NA	NA	149 ± 96	61 ± 38	0.003 ^c^
Viral Load (Log10 copy/mL)	NA	1.17 × 10^3^	4.65 × 10^4^	6.21 × 10^4^	0.02 ^a^
Serotype (*n*)					
DENV-1	NA	8 (72.73%)	10 (76.9%)	7 (87.5%)	
DENV-2	NA	2 (18.18%)	0 (0%)	1 (12.5%)	
DENV-4	NA	1 (9.09%)	2 (15.4%)	0 (0%)	
Undetermined		0 (0%)	1 (7.7%)	0 (0%)	
Type of Infection (*n*, %)					
Primary Infection	NA	3 (27.3%)	3 (23.1%)	0 (0%)	
Secondary Infection	NA	8 (72.7%)	10 (76.9%)	8 (100%)	

HD: healthy controls, ASD: asymptomatic individuals infected with DENV, DF: dengue fever, DHF: dengue hemorrhagic fever. NA: not applicable; ^a^: *p*-values were calculated using ANOVA test. ^b^: *p*-values were calculated using Chi-square test. ^c^: *p*-values were calculated using Mann–Whitney test.

## Supplementary Materials

The following are available online at https://www.mdpi.com/2076-0817/9/12/1060/s1, Supplementary Figure S1. Analysis of autoantibody microarray data. Supplementary Figure S2. The graphs show the *p*-values in the multiple *t*-test comparisons of IgM or IgG autoantibodies in healthy controls compared with those infected with DENV. Supplementary Figure S3. The graphs show the *p*-values in the multiple *t*-test comparisons of IgM or IgG autoantibodies in primary individuals infected with DENV compared with those in secondary individuals infected with DENV. Supplementary Figure S4. The graphs show the *p*-values from multiple *t*-test comparisons of IgM or IgG autoantibodies in ASD compared with those in DF/DHF patients. Supplementary Figure S5. The graphs show autoantibody expression in DF and DHF patients with secondary DENV infection.
