# Supplementary material for: Autoantibody Profiling in Plasma of Dengue Virus–Infected Individuals
Source: Pathogens. 2020 Dec 18;9(12):1060. doi: 10.3390/pathogens9121060 (PMC7766539; doi:10.3390/pathogens9121060)
Supplement: Supplementary file 1 [file pathogens-09-01060-s001.pdf]

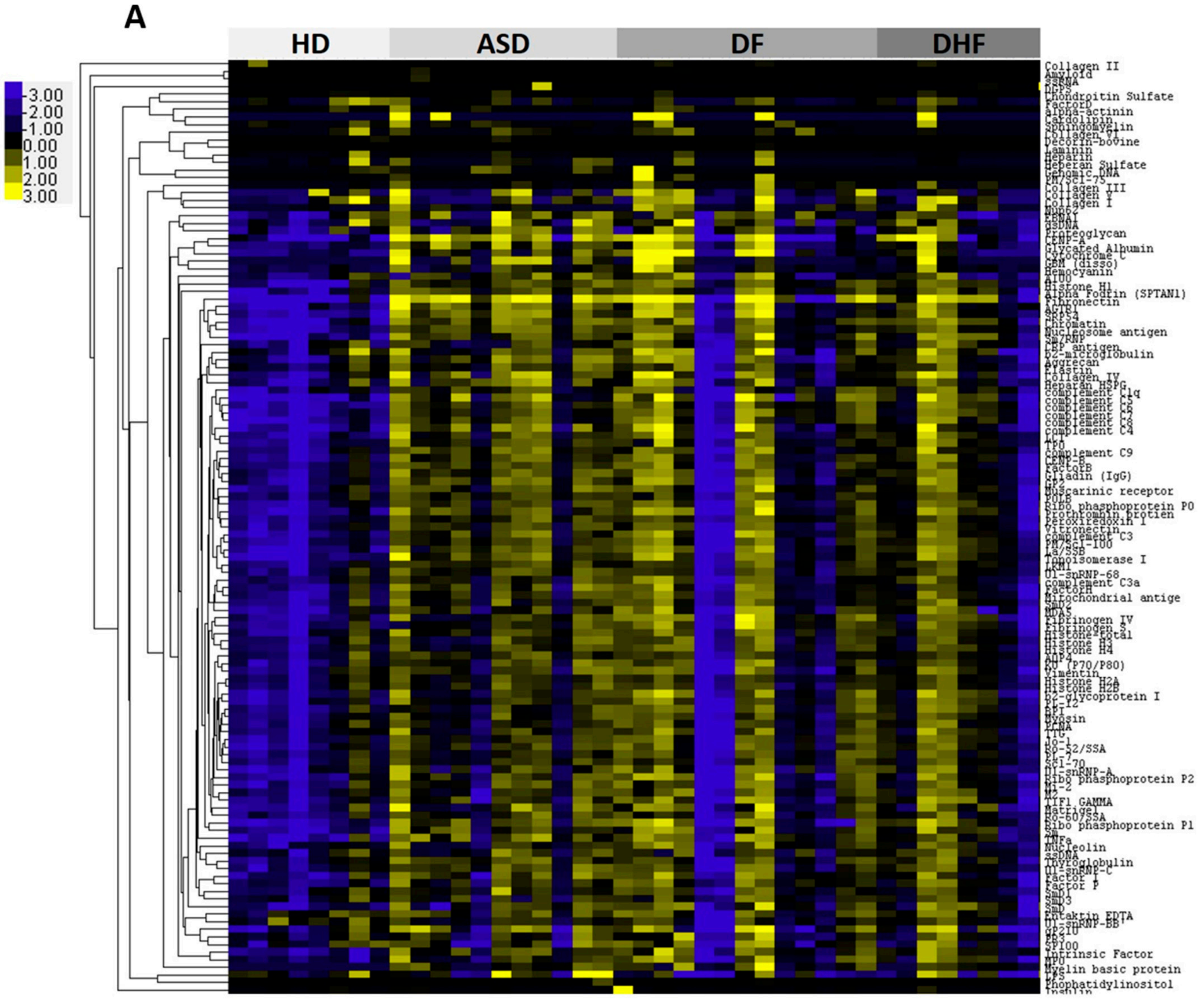
A
HD
ASD
DF
DHF
-3.00
-2.00
-1.00
0.00
1.00
2.00
3.00
Collagen II
Chondroitin Sulfate
alpha-actinin
Decorin-bovine
Heperan Sulfate
Collagen III
Collagen I
dsDNA
Proteoglycan
Glycated Albumin
GBM (disso)
Hemocyanin
Alpha Fodrin (SPTAN1)
Fibronectin
Chromatin
Nucleosome antigen
b2-microglobulin
Aggrecan
complement C1q
complement C4
complement C9
Gliadin (IgG)
Muscarinic receptor
Ribo phasphoprotein P0
complement C3
Topoisomerase I
U1-snRNP-68
complement C3a
Mitochondrial antige
Histone-total
Ku (P70/P80)
b2-glycoprotein I
Myosin
Ro-52/SSA
Ribo phasphoprotein P2
TIF1 GAMMA
Ribo phasphoprotein P1
Nucleolin
Thyroglobulin
Factor P
Entaktin EDTA
Intrinsic Factor
Myelin basic protein
Phophatidylinositol

1

B

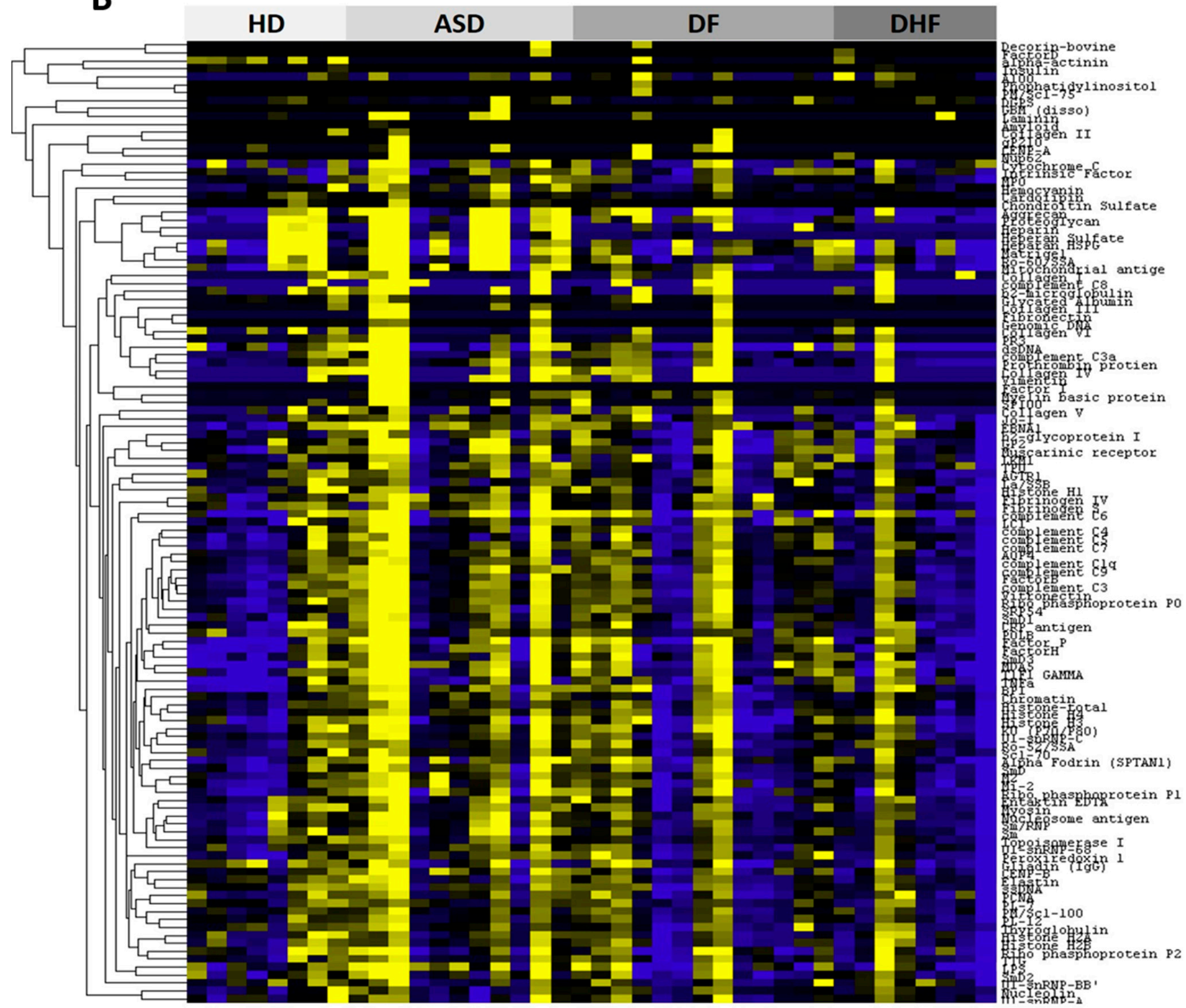
HD
ASD
DF
DHF
Decorin-bovine
alpha-actinin
Insulin
Phosphatidylinositol
GBM (disso)
Collagen II
Cytochrome C
Intrinsic Factor
Hemocyanin
Chondroitin Sulfate
Aggrecan
Proteoglycan
Heparin
Heparan Sulfate
Heparan HSPG
Mitochondrial antige
complement C8
b2-microglobulin
Glycated Albumin
Collagen III
Fibronectin
Genomic DNA
Collagen VI
dsDNA
complement C3a
Prothrombin protien
Collagen IV
Vimentin
Myelin basic protein
Collagen V
P-glycoprotein I
Muscarinic receptor
Histone H1
Fibrinogen IV
complement C6
complement C4
complement C5
complement C7
complement C1q
complement C9
complement C3
Vitronectin
Ribo phosphoprotein P0
CENP antigen
Factor P
FactorH
IFN GAMMA
Chromatin
Histone total
Alpha Fodrin (SPTAN1)
Ribo phosphoprotein P1
Myosin
Nucleosome antigen
Topoisomerase I
Peroxiredoxin 1
Gliadin (IgG)
Elastin
PM/Scl-100
Thyroglobulin
Ribo phosphoprotein P2
U1-snRNP-BB'
Nucleolin

pathogens

MDPI

Supplementary figure S1. Analysis of autoantibody microarray data. Representations are shown of heatmaps of IgM autoantibody (**A**) and IgG autoantibody (**B**). Plasma samples from 34 DENV-infected individuals including ASD (n=11), DF (n=13) and DHF (8) and HD (n=8) were used to probe autoantigen microarrays featuring 123 purified or recombinant autoantigens.

pathogens
MDPI

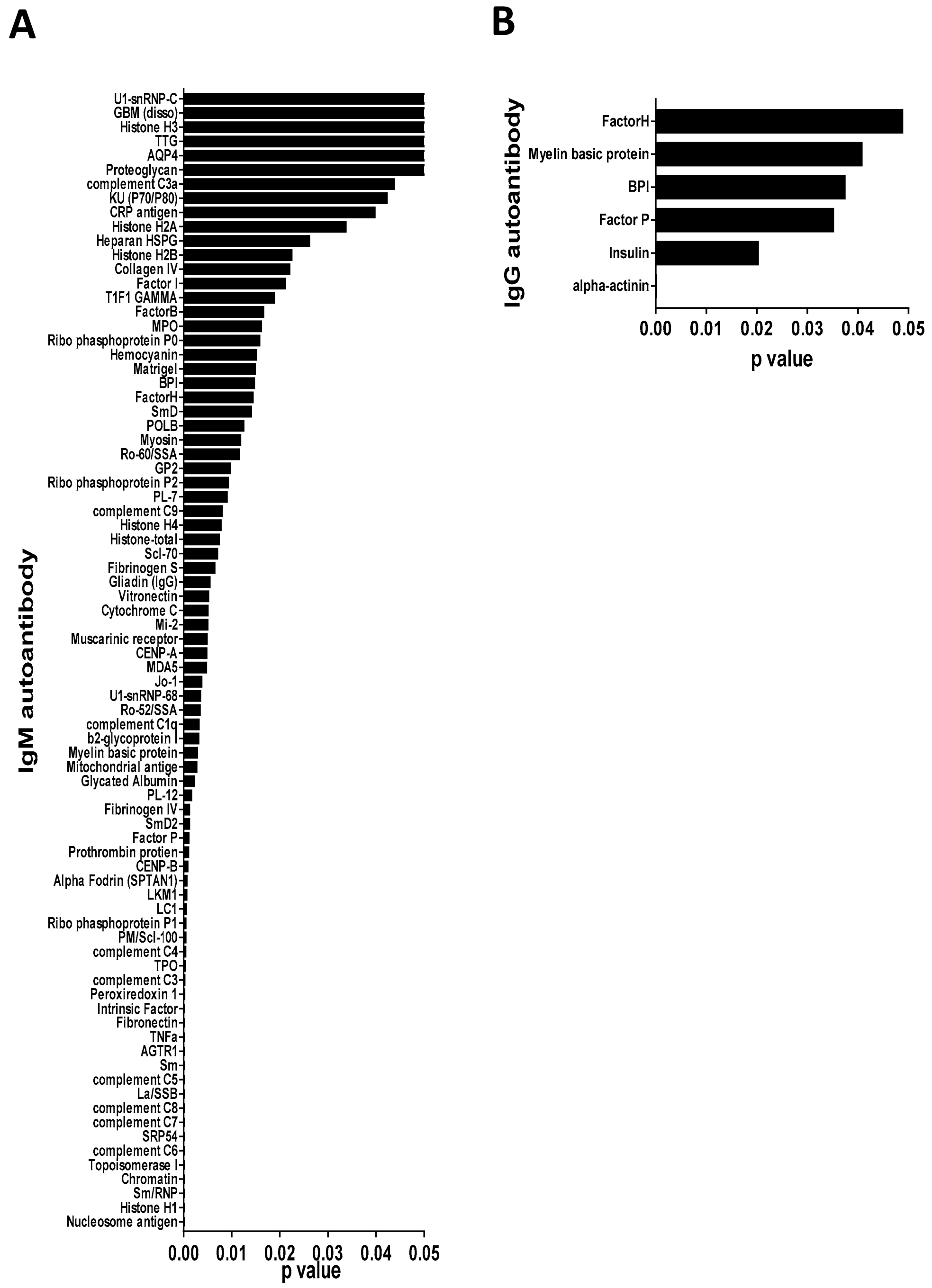

Supplementary figure S2. The graphs show the p-values in the multiple t-test comparisons of IgM (**A**) or IgG (**B**) autoantibodies in healthy controls compared with those in DENV-infected individuals.

pathogens
MDPI

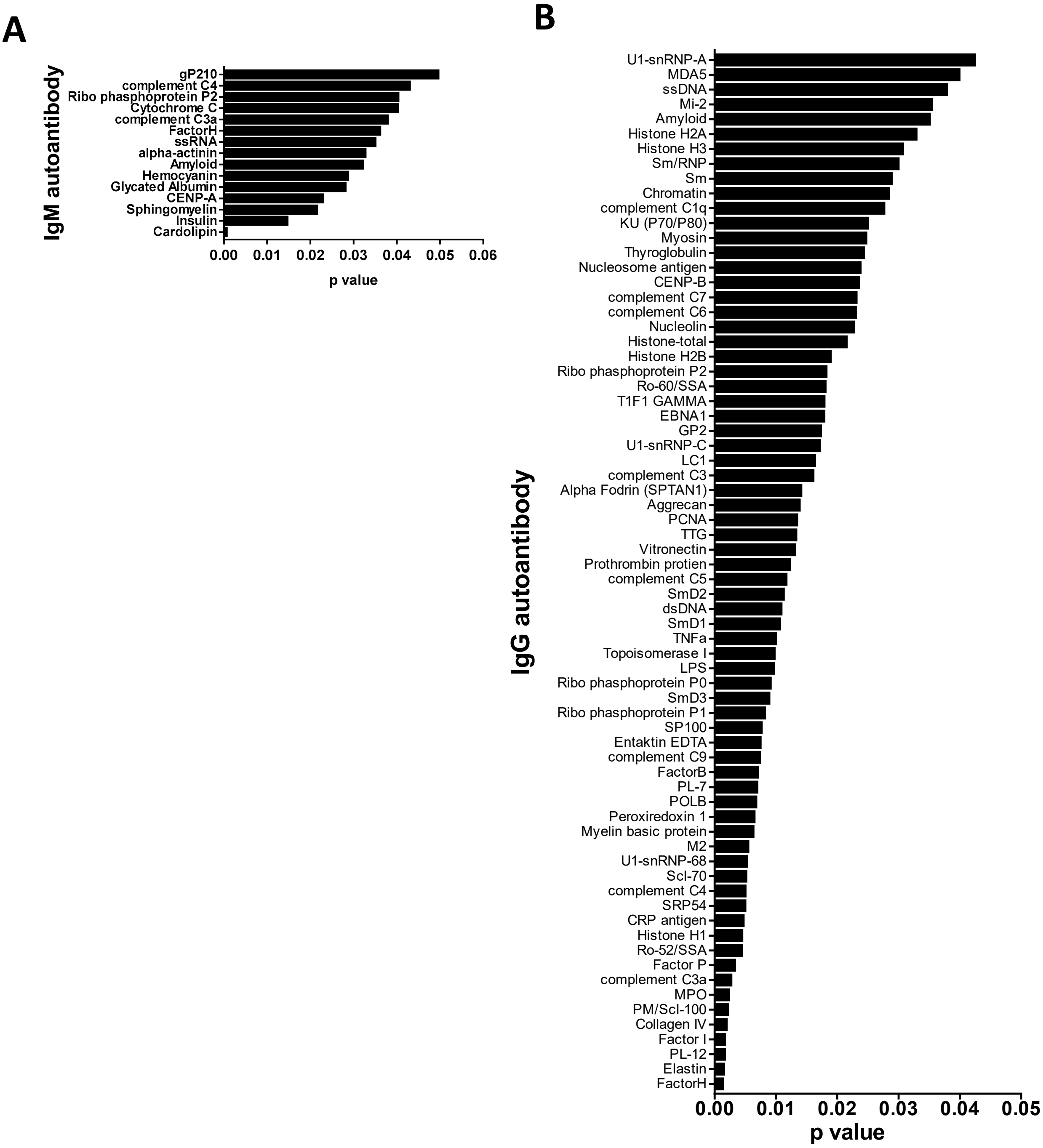

Supplementary figure S3. The graphs show the p-values in the multiple t-test comparisons of IgM (**A**) or IgG (**B**) autoantibodies in primary DENV-infected individuals compared with those in secondary DENV-infected individuals.

pathogens
MDPI

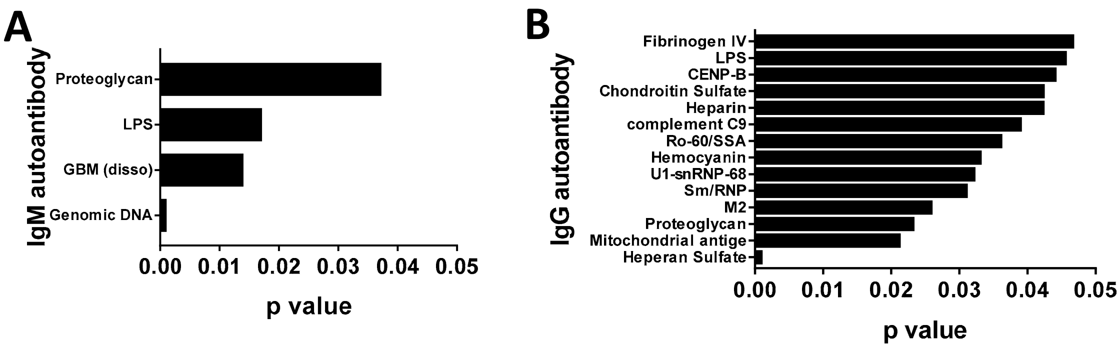

Supplementary figure S4. The graphs show the p-values from multiple t-test comparisons of IgM (**A**) or IgG (**B**) autoantibodies in ASD compared with those in DF/DHF patients.

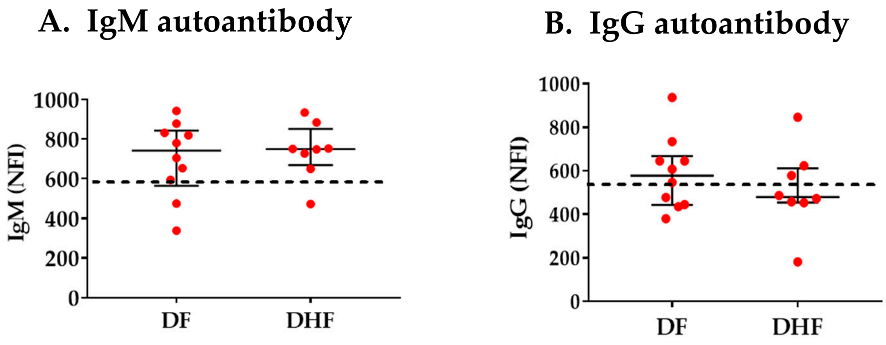

Supplementary figure S5. Autoantibody expression in DF and DHF patients with secondary DENV infection. (**A, B**) Each dot represents the sum of NFI signals of IgM autoantibodies or IgG autoantibodies in each individual. The dashed lines indicate the baseline of the median NFI in HD group. Statistical analysis was done using two-tailed Mann Whitney tests to compare the two groups. Median and interquartile ranges are shown. DF=10, DHF=8.
